# Bust economics: foragers choose high quality habitats in lean times

**DOI:** 10.7717/peerj.1609

**Published:** 2016-01-21

**Authors:** Sonny S. Bleicher, Christopher R. Dickman

**Affiliations:** 1 Department of Ecology and Evolutionary Biology, University of Arizona, Tucson, AZ, USA; 2 School of Biological Sciences, University of Sydney, Sydney, NSW, Australia; 3 Felidae Conservation Fund, Mill Valley, CA, USA

**Keywords:** Dasyurid marsupials, Drought, Foraging games, Giving up density, Habitat selection, Interview chambers, Population dynamics, Sand dunes, Simpson Desert

## Abstract

In environments where food resources are spatially variable and temporarily impoverished, consumers that encounter habitat patches with different food density should focus their foraging initially where food density is highest before they move to patches where food density is lower. Increasing missed opportunity costs should drive individuals progressively to patches with lower food density as resources in the initially high food density patches deplete. To test these expectations, we assessed the foraging decisions of two species of dasyurid marsupials (dunnarts: *Sminthopsis hirtipes* and *S. youngsoni*) during a deep drought, or bust period, in the Simpson Desert of central Australia. Dunnarts were allowed access to three patches containing different food densities using an interview chamber experiment. Both species exhibited clear preference for the high density over the lower food density patches as measured in total harvested resources. Similarly, when measuring the proportion of resources harvested within the patches, we observed a marginal preference for patches with initially high densities. Models analyzing behavioral choices at the population level found no differences in behavior between the two species, but models analyzing choices at the individual level uncovered some variation. We conclude that dunnarts can distinguish between habitat patches with different densities of food and preferentially exploit the most valuable. As our observations were made during bust conditions, experiments should be repeated during boom times to assess the foraging economics of dunnarts when environmental resources are high.

## Introduction

Charles Darwin, upon reaching south western Australia, proclaimed: “…*the soil sandy, and very poor; it supported either a coarse vegetation of thin, low brushwood and wiry grass, or a forest of stunted trees …* [the reader] *will never wish to walk again in so uninviting a country”* (Darwin, 1839: 449–450). Had Darwin visited central Australia instead, he would have found still sandier soils and much harsher and less predictable climatic conditions, but also an extraordinary diversity of small vertebrates that prevail there even during periods of severe rainfall deficit. Many of these species, notably small and medium-sized mammals (Woinarski, Burbidge & Harrison, 2014), have since declined to extinction in central Australia due to changes in land use and the introduction of livestock and exotic species by early European settlers (Dickman et al., 1993; McKenzie et al., 2007). Small carnivorous marsupials are the most successful survivors of these changes and persist throughout the interior deserts of Australia (Dickman, 2003).

The physiological adaptations of desert-dwelling mammals have been much studied (Degen et al., 1997; Geiser, 2004; Schwimmer & Haim, 2009), but the behavioral responses of these mammals to arid environments are less well known. Large species such as giraffe (*Giraffa camelopardalis*) can move long distances to escape regional areas stricken by harsh climatic conditions (Fennessy , 2009), whereas others retreat to localized refuges that provide secure resources and buffered microclimates (McDonald et al., 2015). Reproduction in some large mammals also may slow or cease for months or years to conserve scarce food and water resources (Newsome, 1973). These options are not available for small mammals. Although many species can tolerate prolonged periods of heat by using underground burrows (Roberts, 1972; Richards, 1973; Robert, 1999) and cold by increasing communal huddling (Baudinette, 1972), the lack of food and water resources that accompany extreme climatic conditions necessitate other behavioral responses.

In deserts where food resources fluctuate in a reliable manner between seasons, many small mammals cache food to get them through lean periods and initiate reproduction when food resources increase. Such seasonally predictable behaviors are typical of small mammals in the deserts of North America (Gendron & Reichman, 1995; Van der Merwe, Burke & Brown, 2007; Jorge, Brown & Van der Merwe, 2011), the Sahara and Middle East (Rosenzweig & Sterner, 1970; Kotler et al., 2010), Asia (Shuai & Song, 2011) and South America (Vásquez, Bustamante & Simonetti, 1995). By contrast, in deserts where rainfall is temporally unpredictable and resource pulses exhibit little seasonal reliability, neither food-caching nor seasonal reproduction are likely to be favored. Northern Australian deserts are characterized by highly unpredictable rainfall regimes by world desert standards (Van Etten, 2009). Rodents here reproduce at any time of the year when conditions are favorable (Breed & Leigh, 2011), do not cache food, and can also move relatively long distances (>10 km) to escape areas with depleted resources (Dickman, Predavec & Downey, 1995; Letnic, 2002). Carnivorous marsupials behave similarly, but generally reproduce between winter and the following autumn (Dickman et al., 2001). Caching in these mammals is unknown and would be unexpected owing to the impracticality of storing live or perishable food resources (Gendron & Reichman, 1995).

Uncertainty in when and where food resources will regenerate can be expected to select for dietary opportunism, with individuals utilizing different strategies to ensure resource acquisition and minimize the risk of starvation (Rosenzweig & Sterner, 1970; Davidson, Brown & Inouye, 1980; Brown, 1989). Indeed, Australian desert rodents are generally omnivorous, and eat seeds, green plant material and invertebrates depending on the availability of each food group (Murray et al., 1999). Carnivorous marsupials are, by definition, more constrained to hunting invertebrates or small vertebrates (Fisher & Dickman, 1993a; Fisher & Dickman, 1993b), but nonetheless may track shifts in the local availability of these prey and vary their diets accordingly (Masters, 1998; Bos & Carthew, 2003; Haythornthwaite & Dickman, 2006a). Small (<50 g) and more strictly insectivorous marsupials such as dunnarts (*Sminthopsis* spp.) also appear to forage opportunistically (Dickman, 2014). However, there is some evidence that desert-dwelling dunnarts hunt selectively for certain prey such as lycosid spiders during periods of resource shortage (Sato, 2006; Pena, 2008), perhaps to reduce the potential for intraguild food competition or because these spiders contain water or particular nutrients that dunnarts need during dry times (Chen, Thompson & Dickman, 2004). In addition, larger carnivorous marsupials dominate their smaller counterparts and constrain the types or sizes of prey that they can access (Fisher & Dickman, 1993b).

In this study we assessed the ability of two species of dunnart, captured in central Australia during a prolonged period of rainfall deficit, i.e., a ‘bust,’ to select habitat patches based on their food quality. We asked two questions: (1) Do dunnarts during bust conditions select high quality food patches or forage opportunistically? (2) Is the stronger competitor (larger species) more selective of high quality food patches than the weaker competitor?

Bust periods are characterized by low populations of small mammals and impoverished food resources. We placed individual dunnarts into an ‘interview chamber’ and used optimal patch use theory (Brown, 1988) to gauge their perception of three manipulated habitats (patches) in accordance with Bleicher (2012) and Bleicher (2014). Given the bust conditions that prevailed, we expected dunnarts to ignore the low density patches, where foraging effort would not yield a valuable energetic return, and favor the high density patches instead. As the high density patches are depleted, the difference in value between the high and the lower density patches shrinks. We predicted that, given enough time, the dunnarts would deplete both high and medium-density food patches to the same level. As the two species of dunnart differ in size, and hence probably in competitive ability (Dickman, 1991; Fisher & Dickman, 1993b), we also expected the larger species to be more responsive to variation in patch quality than the smaller species; larger dunnarts should be more selective as they are accustomed to gaining priority of access at food sources, and also have greater food requirements.

## Methods

### Study species and environment

The hairy-footed dunnart (*Sminthopsis hirtipes*, ∼17 g) and the lesser hairy-footed dunnart (*S. youngsoni*, ∼9 g) (Marsupialia, Dasyuridae) occur commonly in sandy habitats in central Australia. Herein, the dunnarts were studied on Ethabuka Reserve, western Queensland (S23°46′, E138°28′), on the eastern edges of their respective geographical ranges. The animals were trapped using 528 pitfall traps set on long term ecological monitoring grids (Dickman et al., 2001; Dickman et al., 2014). For this experiment, each pitfall was open for three consecutive nights, a total of 1,584 trap-nights (number of traps × nights open), with all traps checked at sunrise each morning. Captured animals were weighed and inspected for reproductive condition and then returned to an on-site field laboratory for use in interview chamber experiments (see below). No animals were harmed during this research and all were released back at their capture location within 48 h of capture. While in captivity, the animals were maintained in separate terrariums (15 cm × 20 cm × 20 cm deep) provided with a sandy substrate and vegetation for cover, and kept in quiet, sheltered conditions. Each individual was fed 15 mealworms per day (which ensured they were hungry by the evening) and provided with a petri-dish of water. Post-experiment, all individuals were marked using unique ear clips (to track population dynamics and avoid reuse) before being returned to the site of capture at dawn (Appendix S1).

All work was carried out in April 2015; only 87.3–156.6 mm of rain fell in each of the three years prior to the study compared with the long term annual average of 199.8 mm, with the region suffering ‘severe’ to ‘serious’ rainfall deficiency (lowest 5–10% of records) over this time (data from the nearest town, Bedourie: http://www.bom.gov.au/qld/, accessed 6 June 2015).

### Interview chambers

Two interview chambers were constructed in concordance with Bleicher (2014). Each chamber was constructed from a 30 cm diameter bucket (as a nest box) attached by 5 cm diameter, 30 cm long PVC tubing to three black plastic storage bins (rooms) 66 × 45 × 27 cm high (Appendix S2). Each room was equipped with a round 30 cm diameter tray, filled with one liter of sand, and each tray was set with a different density of live mealworms (*Tenebrio molitor*) that burrowed in the sand: low density (3 mealworms per tray), medium density (6 mealworms) or high (9 mealworms). After dusk, a single dunnart was placed in the nest box and had access to each of the different food-density trays via the PVC tubing. Each dunnart was allowed two hours to forage, following the protocol of Bleicher (2012), and the expectation that this allowed sufficient time for dunnarts to move between and forage in the different food-density trays.

Brown (1988) stated that an animal foraging in a patch will quit harvesting when the costs associated with resource-harvesting coupled with the costs associated with predation risk equal the energetic value of the patch as perceived by the forager. In this experiment, dunnarts were “asked” to compare patches of different initial value in the absence of predation risk. As the dunnart depletes a patch, the diminishing returns render other patches more valuable (a missed opportunity cost). The difference in missed opportunity costs drives animals across the landscape (between rooms of the chamber) examining and comparing patches (Smith & Brown, 1991; Morris & Mukherjee, 2007; Berger-Tal & Kotler, 2014). At the end of each round the amount of resources the forager did not use in the patch due to the aforementioned costs is the giving up density.

We ran up to five rounds (of two hours each) per night based on the availability of dunnarts from the trapping grids between the hours of sunset and sunrise when dunnarts are active. Each individual dunnart was “interviewed” for two consecutive nights (*cf.*Bleicher, 2012) to adjust for holding conditions in the field, predominantly the high daytime temperatures. At the end of each round the animal was removed and returned to its holding container, and fed with extra mealworms. Each of the trays (patches) was sieved and the number of remaining live mealworms recorded to obtain the giving up density. To avoid the possibility of directional bias in selection of patches by the dunnarts, we positioned the density treatment trays in different cardinal directions in each system. The systems were reset after each 2-hour round with fresh mealworms and the next round run with a new dunnart.

Over the period 6–14 April 2015, 18 adult dunnarts were run through the interview chambers. Three further animals went into torpor, and another escaped. The torpid animals were released the following morning when they had resumed normal activity under cover within 10 m of the pitfall trap where they had been captured, and the other 18 animals were released similarly at their capture sites after their second night. Of the 18 animals used, eight were *S. hirtipes* and 10 were *S. youngsoni*. To remove all environmental (i.e., temperature), temporal (i.e., round of the night), directional (i.e., cardinal direction) and individual system effects, we calculated averages per individual within each of the food density treatments (*cf.*Bleicher, 2012; Bleicher, 2014).

### Data analysis

We used two different methods to determine whether the dunnarts selected patches based on the initial density of resources. First, we ran a generalized linear model (GLM) using the number of mealworms consumed during a round as the dependent variable. We also ran a GLM using the proportion of mealworms consumed in the patch as the dependent variable, normalizing the data using an arc sin* square root transformation following Brown et al. (1988) and Ovadia et al. (2005). We ran both models to mitigate the “built in” bias towards the high density patches: more mealworms can be harvested in the high quality patches; however, the same number of mealworms, when harvested in lower density patches represents a greater proportion of the total resources than when harvested in the high density patches. Each GLM used three independent variables, the initial density (ID), the individual dunnart code, and the interaction of species with the initial density.

The second method was used to remove the effect of individuality. We ran two Friedman’s tests of concordance, comparing the way in which each dunnart ranked the importance of the patch’s initial density. We ranked from 1 to 3 the amount of resources used in each treatment (3 being the highest number of mealworms consumed, or the patch with the highest proportion harvested). If the animals concurred on the order, Friedman’s multiple comparison tests were added to the test of concordance to determine the significance of differences between the treatments.

### Ethics

Experimentation procedures were approved by the University of Sydney Animal Ethics Committee (approval number 2013/5297). Scientific field experimentation permits were issued by the Queensland Department of Environment and Heritage Protection for research on native wildlife (Permits WISP15192414 and WITK15192414). Bush Heritage Australia provided access to Ethabuka Reserve where the research was conducted.

## Results

### Trapping success

Thirteen individual rodents were captured in addition to the 22 dunnarts in 1,584 trap-nights (number of traps × nights open), yielding an overall rate of trap success of 2.2% and a success rate of 1.4% for dunnarts alone.

### Foraging responses in habitat patches

**Figure 1:**
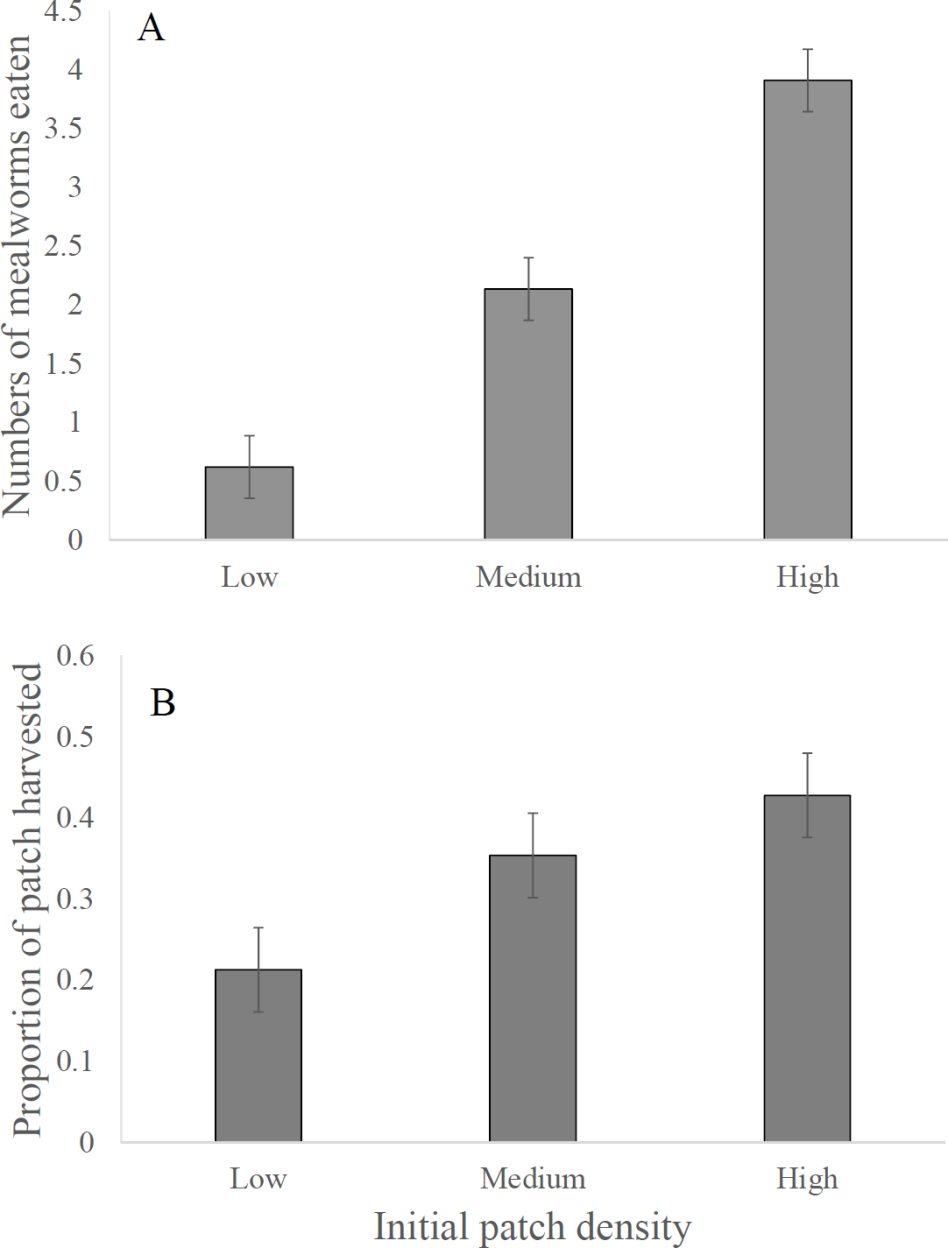
Foraging activity in dunnarts based on initial resource density. Numbers of mealworms eaten by dunnarts in interview chamber experiments providing animals with different initial densities of mealworms, expressed as (A) mean (±SE) numbers eaten, and (B) mean (±SE) proportions of mealworm numbers eaten. Low, low initial food density (3 mealworms per patch); medium, medium initial food density (6 mealworms per patch); high, high initial food density (9 mealworms per patch).

The two models, the first with proportion of patch harvested and the second with number of mealworms consumed as the dependent variables, had relatively high explanatory power, with multiple *R*^2^ values of 0.594 and 0.780, respectively. Neither model showed a difference in patch selection between the dunnart species (Table 1). Both species preferred harvesting in the high food density patches over the low density patches as shown both by the total number of mealworms eaten and proportion of the patch harvested (Table 1, Figs. 1A and 1B). *Post hoc* Tukey Tests of Honestly Significant Difference (THSD) showed pairwise comparisons to be significant for both the total number of mealworms consumed and the proportion harvested, with *p*-values of <0.001 and 0.017, respectively. The difference between the high and medium density patches was significant for total mealworms consumed, but not for the overall proportion of patch harvested, with THSD *p*-values of <0.001 and 0.587, respectively. Similar patterns appeared for the difference between low and medium density patches, with THSD *p*-values of 0.150 for proportion harvested and 0.001 for total mealworms consumed.

**Table 1:** ANOVA tables for general linear models. ANOVA tables for ArcSin*square root proportion of patch harvested and for total mealworms harvested by dunnarts, examining the effects of initial food density of patches (ID), individuality of the dunnart, the interaction between species and the initial food density.

	Variable	Type III SS	df	Mean squares	*F*-ratio	*p*-value
Proportion harvested	Initial density (ID)	0.426	2	0.213	4.391	0.021
	Individual	1.568	17	0.092	1.903	0.057
	Species*ID	0.198	2	0.099	2.04	0.147
	Error	1.551	32	0.048		
Total worms foraged	Initial density	96.272	2	48.136	38.159	<0.001
	Individual	44.667	17	2.627	2.083	0.036
	Species*ID	0.272	2	0.136	0.108	0.898
	Error	40.367	32	1.261

Individual dunnarts responded differently to the food patches, particularly with respect to the number of mealworms consumed, and marginally with respect to the proportion of patches harvested (Table 1). Friedman’s tests of concordance were performed with initial density as the grouping factor, and these revealed that all dunnarts foraged similarly. Using the proportion of patch harvested as the dependent variable, with 2 *d.f.*, ${\chi }_{f}^{2}=1 0.1 8 2$, *p* = 0.006, and the Kendall Coefficient of Concordance (KCC) = 0.283. Using mealworms consumed as the dependent variable resulted in even stronger concordance, with ${\chi }_{f}^{2}=3 0.5 5 4$, *p* < 0.001 and KCC = 0.849.

As shown by Friedman’s multiple comparison test, dunnarts preferred the high density food patches and showed least preference for the low density patches (*p* = 0.001 and <0.001 for proportion harvested and mealworms consumed, respectively). Both models agree that the medium density is also preferred over the low density (*p* = 0.022 and <0.001, respectively). In contrast, the models differed in response to the relationship between medium and high density. Based on the proportion harvested, there was no difference between these initial densities (*p* = 0.239), but a difference was evident based on mealworms consumed (*p* < 0.001).

## Discussion

The small mammal numbers that we recorded here were at least 20-fold lower than they had been four years previously during a period of above-average rainfall (Dickman et al., 2014), confirming our assessment that this study was carried out during a significant population bust. The two species of dunnart behaved as we had predicted during the bust conditions by foraging preferentially in high density rather than low density food patches. However, some findings were not anticipated: the two species responded similarly to the different food patches, and there also appeared to be some individual variation in response to food. We explore these responses further below.

### Patch selection

Both species preferred foraging in the higher food density patches, supporting the hypothesis that when resources are low foragers will not expend much effort in exploiting low quality patches. The preference of dunnarts for the high density patches (Fig. 1) suggests that animals assessed and responded predictably to variation in patch quality. Indeed, at the end of most 2-hour rounds, footprints were visible in the sand of every food tray, suggesting that dunnarts had visited each tray but then elected to focus their foraging principally where food density was highest. An alternative expectation is that dunnarts might have behaved more opportunistically, foraging wherever they visited first and employing a “beggar-not-chooser” strategy (Smith & Brown, 1991). In times of hardship any resource should be attractive (Haythornthwaite & Dickman, 2006b) and, indeed, we recorded four individual *S. youngsoni* that ate equal numbers of mealworms from each food-density treatment. However, as a whole, animals foraged principally in the high density patch, and most likely did so after inspecting and assessing the value of each patch. Dunnarts can move hundreds of meters while foraging at night (Haythornthwaite & Dickman, 2006a; Haythornthwaite & Dickman, 2006b) and often return to particular patches to assess their value (Sato, 2006). Inspection of each of the trays in the interview chambers would have allowed animals to assess their relative benefits and focus on the high density patches, while also minimizing missed opportunity costs. The preference for high density food patches was most obvious for the total number of mealworms eaten; the weaker preference for the higher initial food density in terms of the proportion of resources harvested from the patches perhaps reflected dunnarts moving from the high to lower value patches as the marginal value of the high density food patches fell.

### Species-level and individual responses

Both species responded in a similar manner to the resource variation between the patches, in marked contrast to the hypothesis that the stronger competitor would be more selective than the small competitor. Two explanations can be advanced.

Firstly, although we had expected *S. hirtipes* to respond most strongly to variation in patch quality, this expectation was based on the assumption that it would have competitive priority of access to shared resources and thus increase the apprehension of *S. youngsoni* when foraging in high quality patches. In general, interspecific competition should increase during bust conditions as resources deplete and congeners expand their search areas into each other’s preferred foraging territories (Newell et al., 2014). However, if the population sizes of competitors also decrease so that *per capita* resource availability remains constant (Fretwell, 1972), the species may segregate into preferred habitats and frequencies of interspecific encounters then will be low. Examples of this ‘rarefaction’ effect can be found in Negev Desert gerbils where competition decreases with decreasing population size (Abramsky, Ovadia & Rosenzweig, 1994) and gerbils show segregation by the substrate in which they forage (Kotler et al., 2001). Similarly, in granivorous communities in the Mojave, Sonoran and Chihuahuan Deserts, rodent species segregate predominantly based on their ability to manage predation risk as evident in their habitat selection (Rosenzweig, 1973; Lemen & Rosenzweig, 1978; Davidson, Inouye & Brown, 1982; Kotler, 1984; Longland & Price, 1991) and dietary choices (Davidson, Brown & Inouye, 1980).

In our study, it is possible that the *S. youngsoni* we used had seldom encountered their larger congeners due to their low population size, and hence had little apprehension about the potential for conflict with them in the high density food patches. Alternatively, it is possible that the novel but relatively neutral environments of the interview chambers provided no cues about potential competitors (or predators), allowing both species of dunnarts to assess and exploit the food patches simply on what they offered.

Secondly, we had also expected that *S. hirtipes* would focus on the high density food patches to procure food most effectively in order to maintain its relatively large body size. However, this need could have been reduced if animals had entered short periods of torpor so as to reduce their energy needs. This phenomenon occurs commonly in many small mammal species, such as pocket mice of the southwestern US (Brown & Zeng, 1989). Similarly, many dasyurid marsupials can enter short-term torpor to save energy (Geiser, 2004), and are more likely to do so if their diet comprises mostly invertebrates rather than other vertebrates (Pavey et al., 2009). However, we do not consider this to be a compelling explanation for our results: *S. hirtipes* rarely eats vertebrates (Fisher & Dickman, 1993b), and no animals were found to be torpid at the end of their 2-hour trial in the interview chambers.

The variance in response between individuals, some not discerning between the medium and high density patches (seen in the Friedman’s test of concordance for proportion harvested), suggests that the population may have varied behavioral strategies. Some individuals are very selective, and others opportunistic. This variation is unlikely to have arisen due to age or reproductive differences. Although we used both sexes of the two dunnart species in our experiments, all were adults and none was in reproductive condition; the breeding season for both species would likely have concluded months earlier during summer (Dickman et al., 2001). Different species of insectivorous mammals show varied foraging strategies: from selection of prey that yields high rates of energy return (Calver, Bradley & King, 1988), through habitat patches that differ in the likelihood of providing adequate food rewards (Brown & Munger, 1985), to shifts in prey preference as individuals learn (Dickman, 1988). It is possible that the dunnarts we observed had individually different foraging strategies, but further work is needed to confirm this.

Overall, our results are consistent with the notion that dunnarts can distinguish between habitat patches with different densities of food, and that it is advantageous for them to do so during bust conditions when environmental resources are depleted. We would predict them to be less selective of high density food patches when conditions are more productive owing to the greater abundance of food resources that would be available then, and suggest that this expectation be tested by repeating the experiments after rain when environmental resources have regenerated.

### Prospectus

This experiment was run as a pilot for the use of interview chambers with Australian fauna, and for adapting the chambers to work with insectivorous foragers. The results showed that the versatility of these chambers is great, and the number of experiments that may be run with them equally so. The necessity for replicating the experiments under boom conditions has been mentioned above, and work is already planned for a year when these conditions arise. In laboratory conditions, these chambers may be used in combination with RFID pit tags that are installed subcutaneously on the animals and may be used to track their movements and also reveal the amount of time that animals spend in different foraging patches. Experiments that test the effect of competition between these (and other) species will also highlight whether bust conditions amplify the impact of inter-species (and intra population) interactions. Although the interview chambers provided results that were consistent with our understanding of how animals forage when unconstrained in the field (Sato, 2006), we emphasize that care should be taken to ensure that interview chamber results generally do reflect study species’ natural foraging behaviors. We intended to use the interview chamber system to further study predator–prey interactions in the Simpson Desert, but were unable to trap enough predators for the planned experiments during the current season with resources being at very low levels. We invite, and encourage, our colleagues to adopt this simple (and low cost) experimental set up, test its limits and devise new uses for it.

## Supplemental Information

10.7717/peerj.1609/supp-1 Appendix SI *Sminthopsis hirtipes* handled in terrarium before release

10.7717/peerj.1609/supp-2 Appendix SII Picture of interview chamber systems in the field at Ethabuka Reserve at the start of an experimental round

10.7717/peerj.1609/supp-3 Data S1 Data for interview-chambers using dunnarts at Ethabuka QLD, April 2015 Raw data collected during the habitat quality experiment for two dunnart speceis in the Simposon Desert during the Fall Sampling Season, April 2015.

## Additional Information and Declarations

### Field Study Permissions

The following information was supplied relating to field study approvals (i.e., , approving body and any reference numbers): Scientific field experimentation permits were issued by the Queensland Department of Environment and Heritage Protection for research on native wildlife (Permits WISP15192414 and WITK15192414). Bush Heritage Australia provided access to Ethabuka Reserve where the research was conducted.

### Data Availability

The following information was supplied regarding data availability: Data can be found at: https://sonnybleicher.wordpress.com/data-repository-for-open-source-manuscripts/.
