# Supplementary figures and images for: Bust economics: foragers choose high quality habitats in lean times
Source: PeerJ. 2016 Jan 21;4:e1609. doi: 10.7717/peerj.1609 (PMC4734440; doi:10.7717/peerj.1609)

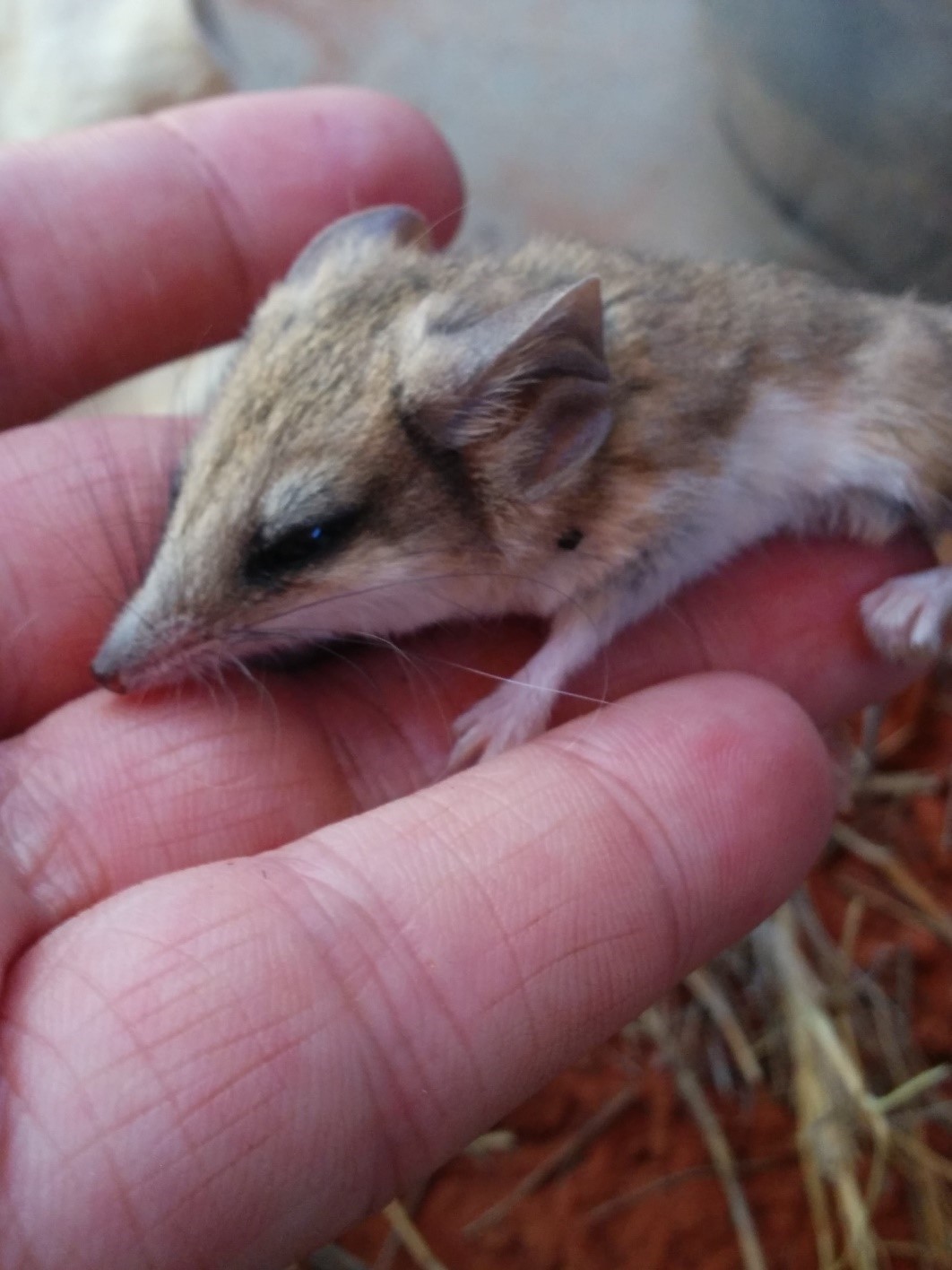

Supplement: Appendix SI [file peerj-04-1609-s001.jpg]

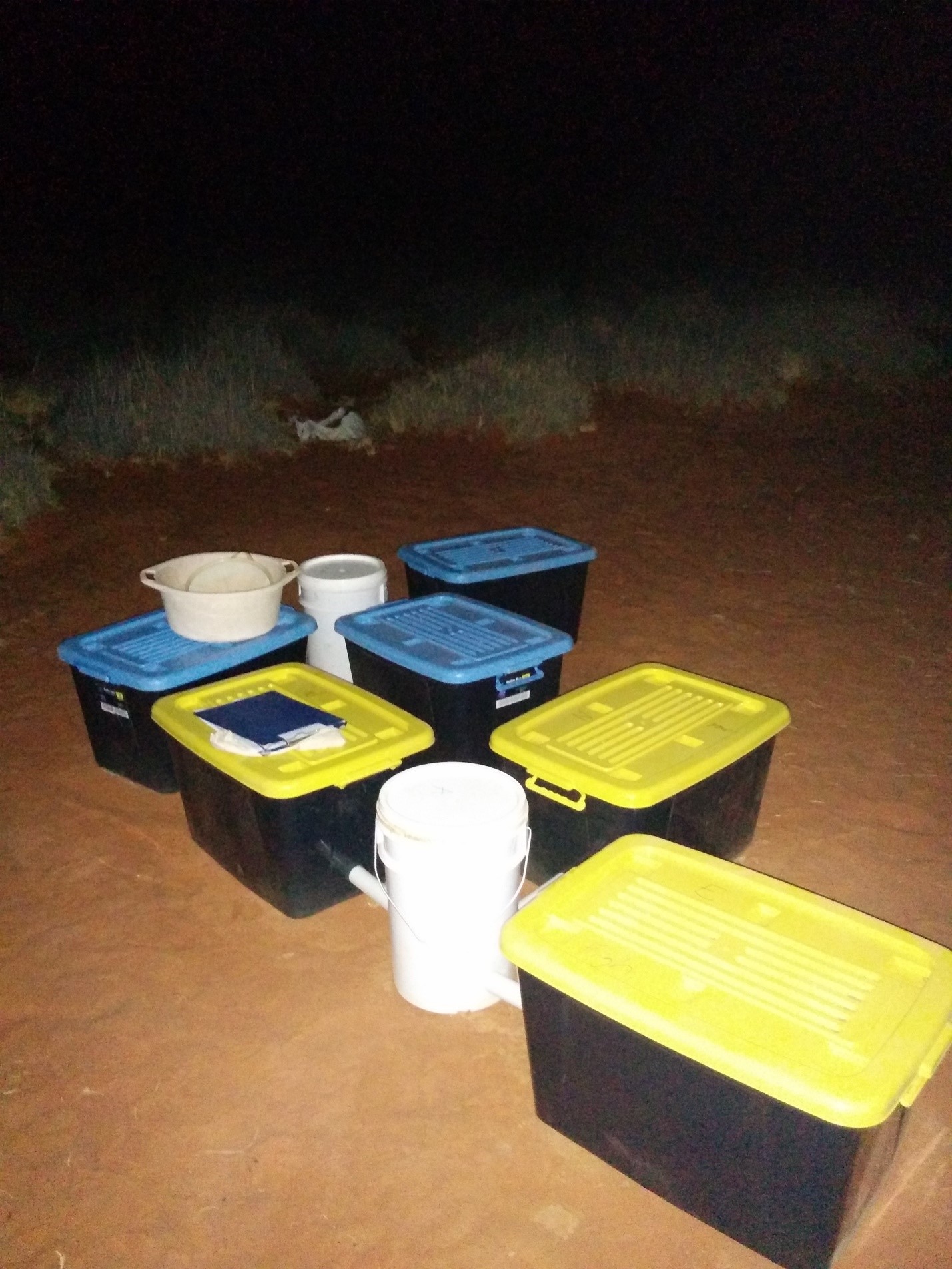

Supplement: Appendix SII [file peerj-04-1609-s002.jpg]
