# Supplementary material for: Bust economics: foragers choose high quality habitats in lean times
Source: PeerJ. 2016 Jan 21;4:e1609. doi: 10.7717/peerj.1609 (PMC4734440; doi:10.7717/peerj.1609)
Supplement: Data S1 — Raw data collected during the habitat quality experiment for two dunnart speceis in the Simposon Desert during the Fall Sampling Season, April 2015. [file peerj-04-1609-s003.pdf]

DATE	ROUND	SYSTEM	CARDIANL	TREATMENT	SPECIES	ID	INITIAL DENSITY	GUD
6-Apr-15	1	A	W	LOW	SH	BSH0504	3	3
6-Apr-15	1	A	N	MEDIUM	SH	BSH0504	6	4
6-Apr-15	1	A	E	HIGH	SH	BSH0504	9	8
6-Apr-15	1	B	N	HIGH	LD	2SY0404	9	6
6-Apr-15	1	B	E	LOW	LD	2SY0404	3	3
6-Apr-15	1	B	S	MEDIUM	LD	2SY0404	6	6
6-Apr-15	2	A	W	LOW	LD	BSY0504	3	3
6-Apr-15	2	A	N	MEDIUM	LD	BSY0504	6	6
6-Apr-15	2	A	E	HIGH	LD	BSY0504	9	5
6-Apr-15	2	B	N	HIGH	LD	ISY0604	9	6
6-Apr-15	2	B	E	LOW	LD	ISY0604	3	3
6-Apr-15	2	B	S	MEDIUM	LD	ISY0604	6	5
5-Apr-15	2	A	W	LOW	LD	2SY0404	3	3
5-Apr-15	2	A	N	MEDIUM	LD	2SY0404	6	3
5-Apr-15	2	A	E	HIGH	LD	2SY0404	9	4
5-Apr-15	2	B	N	HIGH	LD	DSY0504	9	3
5-Apr-15	2	B	E	LOW	LD	DSY0504	3	3
5-Apr-15	2	B	S	MEDIUM	LD	DSY0504	6	2
5-Apr-15	3	B	N	HIGH	SH	BSH0504	9	5
5-Apr-15	3	B	E	LOW	SH	BSH0504	3	3
5-Apr-15	3	B	S	MEDIUM	SH	BSH0504	6	3
7-Apr-15	2	B	N	HIGH	LD	7SY0704	9	6
7-Apr-15	2	B	E	LOW	LD	7SY0704	3	3
7-Apr-15	2	B	S	MEDIUM	LD	7SY0704	6	3
7-Apr-15	3	A	W	LOW	LD	ISY0604	3	3
7-Apr-15	3	A	N	MEDIUM	LD	ISY0604	6	5
7-Apr-15	3	A	E	HIGH	LD	ISY0604	9	8
8-Apr-15	1	A	W	LOW	LD	7SY0704	3	3
8-Apr-15	1	A	N	MEDIUM	LD	7SY0704	6	4
8-Apr-15	1	A	E	HIGH	LD	7SY0704	9	7
8-Apr-15	1	B	N	HIGH	SH	FSH0804	9	6
8-Apr-15	1	B	E	LOW	SH	FSH0804	3	3
8-Apr-15	1	B	S	MEDIUM	SH	FSH0804	6	6
8-Apr-15	2	A	W	LOW	LD	11SY0804	3	2
8-Apr-15	2	A	N	MEDIUM	LD	11SY0804	6	4
8-Apr-15	2	A	E	HIGH	LD	11SY0804	9	5
9-Apr-15	1	A	W	LOW	SH	BSH0804	3	0
9-Apr-15	1	A	N	MEDIUM	SH	BSH0804	6	5
9-Apr-15	1	A	E	HIGH	SH	BSH0804	9	6
9-Apr-15	1	B	N	HIGH	LD	11SY0804	9	5
9-Apr-15	1	B	E	LOW	LD	11SY0804	3	1
9-Apr-15	1	B	S	MEDIUM	LD	11SY0804	6	5
10-Apr-15	1	A	W	LOW	SH	8SH1004	3	3
10-Apr-15	1	A	N	MEDIUM	SH	8SH1004	6	4
10-Apr-15	1	A	E	HIGH	SH	8SH1004	9	4
11-Apr-15	1	A	W	LOW	SH	7SH1104	3	3

11-Apr-15	1	A	N	MEDIUM	SH	7SH1104	6	6
11-Apr-15	1	A	E	HIGH	SH	7SH1104	9	8
11-Apr-15	1	B	N	HIGH	SH	8SH1004	9	5
11-Apr-15	1	B	E	LOW	SH	8SH1004	3	2
11-Apr-15	1	B	S	MEDIUM	SH	8SH1004	6	2
13-Apr-15	1	A	W	LOW	SH	3CSH1304	3	3
13-Apr-15	1	A	N	MEDIUM	SH	3CSH1304	6	1
13-Apr-15	1	A	E	HIGH	SH	3CSH1304	9	5
13-Apr-15	1	B	N	HIGH	SH	4DSH1304	9	3
13-Apr-15	1	B	E	LOW	SH	4DSH1304	3	1
13-Apr-15	1	B	S	MEDIUM	SH	4DSH1304	6	1
13-Apr-15	2	A	W	LOW	SH	4ASH1304	3	3
13-Apr-15	2	A	N	MEDIUM	SH	4ASH1304	6	3
13-Apr-15	2	A	E	HIGH	SH	4ASH1304	9	0
13-Apr-15	2	B	N	HIGH	SH	5ASH1304	9	5
13-Apr-15	2	B	E	LOW	SH	5ASH1304	3	0
13-Apr-15	2	B	S	MEDIUM	SH	5ASH1304	6	6
13-Apr-15	3	A	W	LOW	SY	3BSY1304	3	3
13-Apr-15	3	A	N	MEDIUM	SY	3BSY1304	6	5
13-Apr-15	3	A	E	HIGH	SY	3BSY1304	9	8
13-Apr-15	3	B	N	HIGH	SY	4BSY1304	9	1
13-Apr-15	3	B	E	LOW	SY	4BSY1304	3	2
13-Apr-15	3	B	S	MEDIUM	SY	4BSY1304	6	3
13-Apr-15	4	A	W	LOW	SY	4ASY1304	3	3
13-Apr-15	4	A	N	MEDIUM	SY	4ASY1304	6	4
13-Apr-15	4	A	E	HIGH	SY	4ASY1304	9	2
13-Apr-15	4	B	N	HIGH	SY	3ASY1304	9	1
13-Apr-15	4	B	E	LOW	SY	3ASY1304	3	3
13-Apr-15	4	B	S	MEDIUM	SY	3ASY1304	6	5
13-Apr-15	5	A	W	LOW	SH	8SH1204	3	0
13-Apr-15	5	A	N	MEDIUM	SH	8SH1204	6	1
13-Apr-15	5	A	E	HIGH	SH	8SH1204	9	4
14-Apr-15	1	A	W	LOW	SY	3ASY1304	3	3
14-Apr-15	1	A	N	MEDIUM	SY	3ASY1304	6	3
14-Apr-15	1	A	E	HIGH	SY	3ASY1304	9	7
14-Apr-15	1	B	N	HIGH	SY	4ASY1304	9	7
14-Apr-15	1	B	E	LOW	SY	4ASY1304	3	3
14-Apr-15	1	B	S	MEDIUM	SY	4ASY1304	6	3
14-Apr-15	2	A	W	LOW		4DSH1304	3	1
14-Apr-15	2	A	N	MEDIUM		4DSH1304	6	5
14-Apr-15	2	A	E	HIGH		4DSH1304	9	8
14-Apr-15	2	B	N	HIGH		3CSH1304	9	7
14-Apr-15	2	B	E	LOW		3CSH1304	3	2
14-Apr-15	2	B	S	MEDIUM		3CSH1304	6	5
14-Apr-15	3	A	W	LOW		5ASH1304	3	3
14-Apr-15	3	A	N	MEDIUM		5ASH1304	6	5
14-Apr-15	3	A	E	HIGH		5ASH1304	9	8

14-Apr-15	3	B	N	HIGH	4ASH1304	9	5
14-Apr-15	3	B	E	LOW	4ASH1304	3	3
14-Apr-15	3	B	S	MEDIUM	4ASH1304	6	2
14-Apr-15	4	A	W	LOW	4BSY1304	3	2
14-Apr-15	4	A	N	MEDIUM	4BSY1304	6	5
14-Apr-15	4	A	E	HIGH	4BSY1304	9	6

PROP_HAR	TOTAL_FO	COMMENTS
0	0	
0.333333	2	
0.111111	1	
0.333333	3	
0	0	
0	0	
0	0	
0	0	
0.444444	4	
0.333333	3	
0	0	
0.166667	1	
0	0	
0.5	3	
0.555556	5	
0.666667	6	
0	0	
0.666667	4	
0.444444	4	
0	0	
0.5	3	
0.333333	3	
0	0	
0.5	3	
0	0	
0.166667	1	
0.111111	1	
0	0	
0.333333	2	
0.222222	2	
0.333333	3	
0	0	
0	0	
0.333333	1	
0.333333	2	
0.444444	4	
1	3	3 HOURS
0.166667	1	3 HOURS
0.333333	3	3 HOURS
0.444444	4	3 HOURS
0.666667	2	3 HOURS
0.166667	1	3 HOURS
0	0	
0.333333	2	
0.555556	5	
0	0	UNFORAGED

0	0	UNFORAGED
0.111111	1	UNFORAGED
0.444444	4	
0.333333	1	
0.666667	4	
0	0	
0.833333	5	
0.444444	4	
0.666667	6	
0.666667	2	
0.833333	5	
0	0	
0.5	3	
1	9	
0.444444	4	
1	3	
0	0	
0	0	LATHARGIC
0.166667	1	LATHARGIC
0.111111	1	LATHARGIC
0.888889	8	
0.333333	1	
0.5	3	
0	0	
0.333333	2	
0.777778	7	
0.888889	8	
0	0	
0.166667	1	
1	3	
0.833333	5	
0.555556	5	
0	0	
0.5	3	
0.222222	2	
0.222222	2	
0	0	
0.5	3	
0.666667	2	
0.166667	1	
0.111111	1	
0.222222	2	
0.333333	1	
0.166667	1	
0	0	ESACPED
0.166667	1	ESACPED
0.111111	1	ESACPED

0.444444	4
0	0
0.666667	4
0.333333	1
0.166667	1
0.333333	3
